# Epiphyseal enchondroma masking as osteoid osteoma: a case report

**DOI:** 10.1186/s40001-021-00504-y

**Published:** 2021-05-07

**Authors:** Xuyang Cao, Qiang Ren, Xiangnan Li, Yiren Tian, Zhendong Wang

**Affiliations:** 1 Department of Orthopedics, General Hospital of Jizhong Energy Xingtai Mining Group, Xingtai, 054000 Hebei China; 2 Department of Orthopedics, Hebei Provincial Chidren’s Hospital, No. 133 Jianhua South Street, Chang’an District, Shijiazhuang, 050000 Hebei China; 3 Department of Rehabilitation, Hebei Provincial Academic of Traditional Chinese Medicine, Shijiazhuang, 050000 Hebei China

**Keywords:** Enchondromas originating, Osteoid osteoma, Case report

## Abstract

**Background:**

Enchondromas originating in the epiphyses of long bones are rare and epiphyseal osteoid osteomas are also uncommon. Diagnosis can become elusive when enchondromas or osteoid osteomas occur in atypical locations and present with nonspecific clinical and imaging characteristics.

**Case presentation:**

We report a case of epiphyseal enchondroma of the left proximal femur in a 15-year-old girl with a 2-month history of left lower extremity pain. Preoperative CT displayed thickened cortex in the anterior surface of the left proximal femur with specks of calcification and inhomogeneity of the adjacent bone marrow cavity. She was diagnosed with osteoid osteoma. Postoperative pathological examination of surgically excised specimens revealed a diagnosis of enchondromas.

**Conclusions:**

Our case highlights that enchondroma should be considered in lesions of the epiphysis.

## Background

Enchondroma is a common benign cartilage tumor in children [1] and accounts for approximately 3% of bone tumors and up to 13% of benign bone tumors [2, 3]. Enchondroma is very rare. In a study of 49 children and adolescents with long bone epiphyseal lesions, El-Ali et al. found only 1 (2.0%) case of enchondroma [4]. Potter et al*.* reviewed 761 cases of enchondroma over the duration of 55 years and found that 4.3% of those were epiphyseal enchondromas [1]. The disease has an indolent and subtle clinical course and often does not become manifest until 20 years of age. It has a predilection for short bones, especially the phalanx and metacarpal, and in long bones, enchondroma more frequently occurs in the femur and humerus [1]. Enchondromas originating in the epiphyses of long bones are rare, and frequently prompt patients to seek early medical attention as epiphyseal lesions often produce pain in the absence of trauma or other physical insults [5]. Osteoid osteoma rarely occurs in the epiphysis and is characterized by an osteolytic area surrounded by variable degrees of reactive sclerosis with secondary bone marrow changes that could lead to erroneous diagnoses [1]. Diagnosis can become elusive when enchondromas or osteoid osteomas occur in atypical locations and present with nonspecific clinical and imaging characteristics [6]. Here, we report a case of epiphyseal enchondroma masking as osteoid osteoma of the left proximal femur in a 15-year-old girl with left lower extremity pain.

## Case presentation

A 15-year-old girl presented to our hospital on September 13, 2018 with a 2-month history of pain in the left lower extremity that worsened for 1 month. Major clinical manifestations included lumbago and radiating pain along the femoral nerve distribution with decreased sensation and paresthesia of the left lower extremity. She also had limitation of motion of the lower limb, which was relieved after rest. The patient visited a local hospital in July 2018 and received no definite diagnosis. She was given vitamins and calcium supplementation, but her pain was not alleviated. In August 2018, her pain worsened and swelling also appeared in the left thigh. She visited the Department of Neurology of our hospital on September 6, 2018. Electromyography revealed injury of the left femoral nerve; she received mecobalamin and vitamin B12, but pain failed to improve. The patient had no history of trauma. Her medical history was unremarkable.

**Fig. 1:**
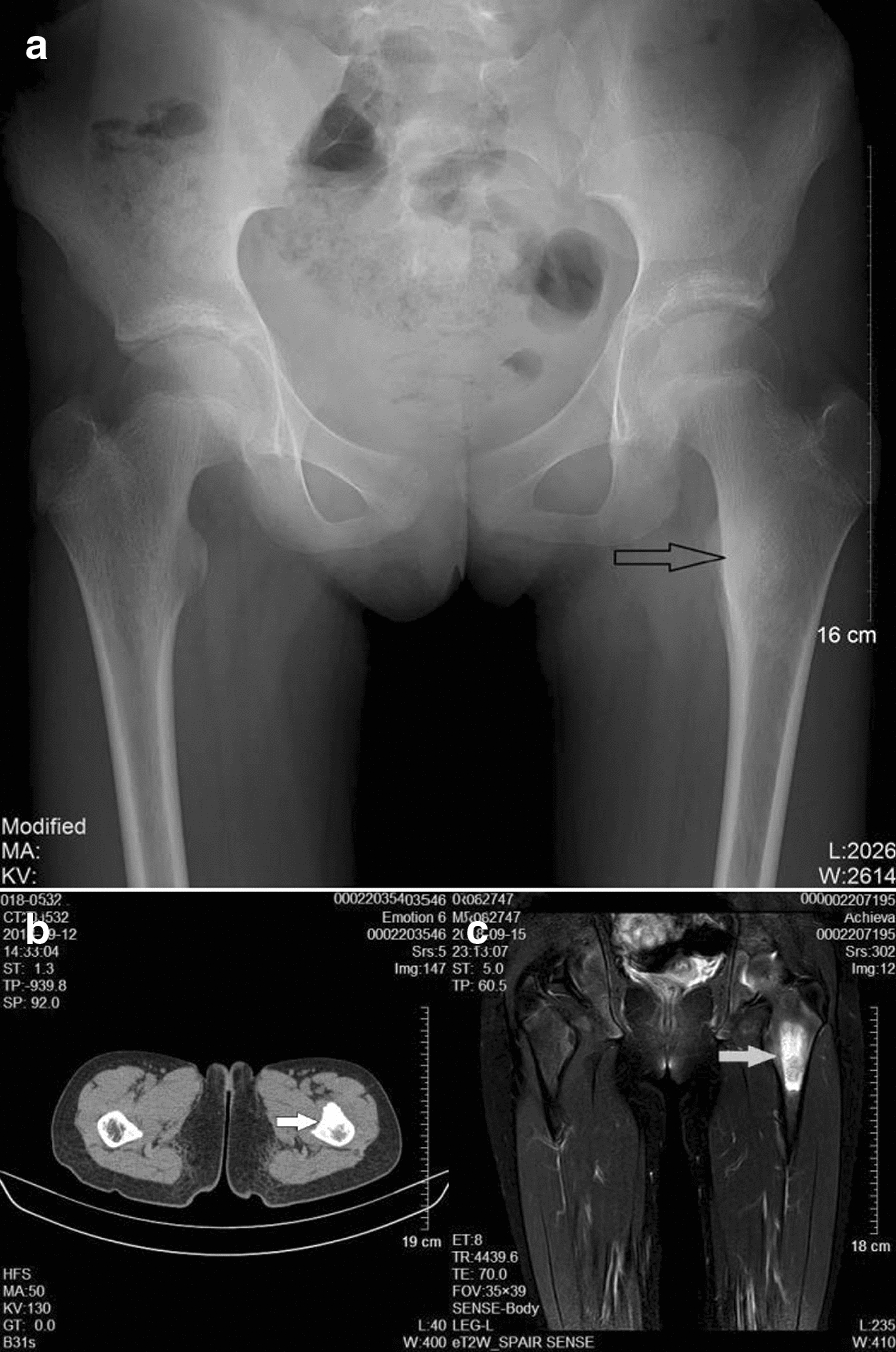
**a** Preoperative X-ray examinations show increased density (arrow) of the greater trochanter of the left femur in a 15-year-old girl presenting with a 2-month history of left lower leg pain. **b**, **c** CT coronal and sagittal image of the lesion (arrow)

**Fig. 2:**
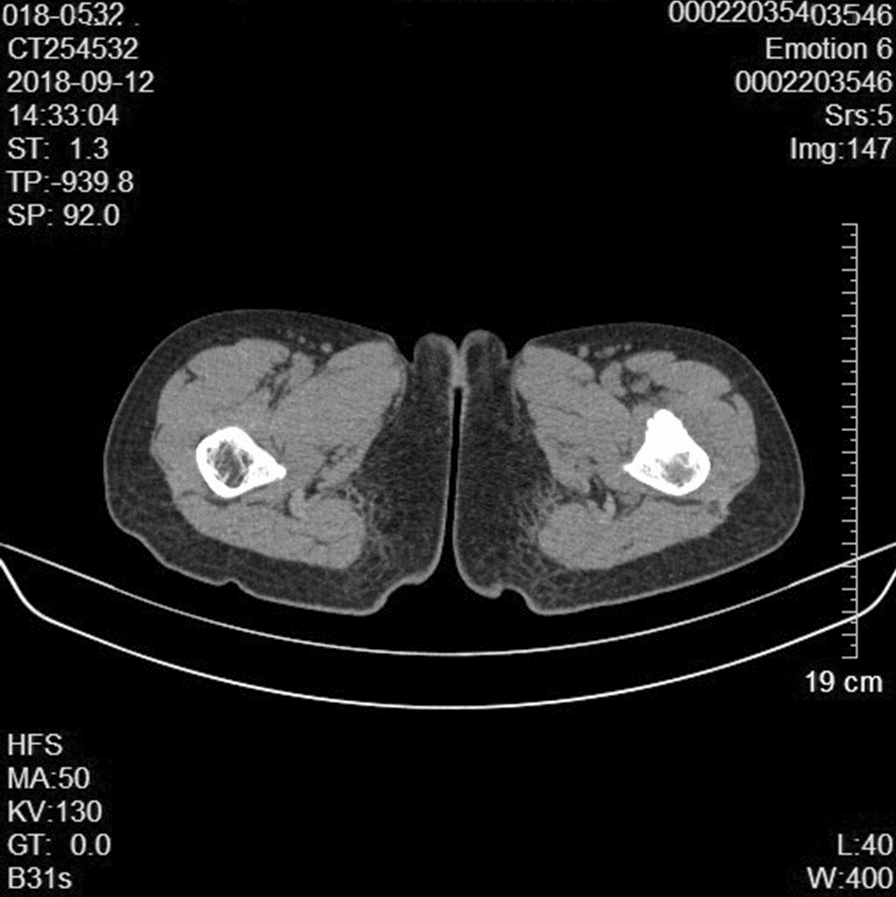
Preoperative CT displays thickened cortex in the anterior surface of the left proximal femur with specks of calcification and inhomogeneity of the adjacent bone marrow cavity

**Fig. 3:**
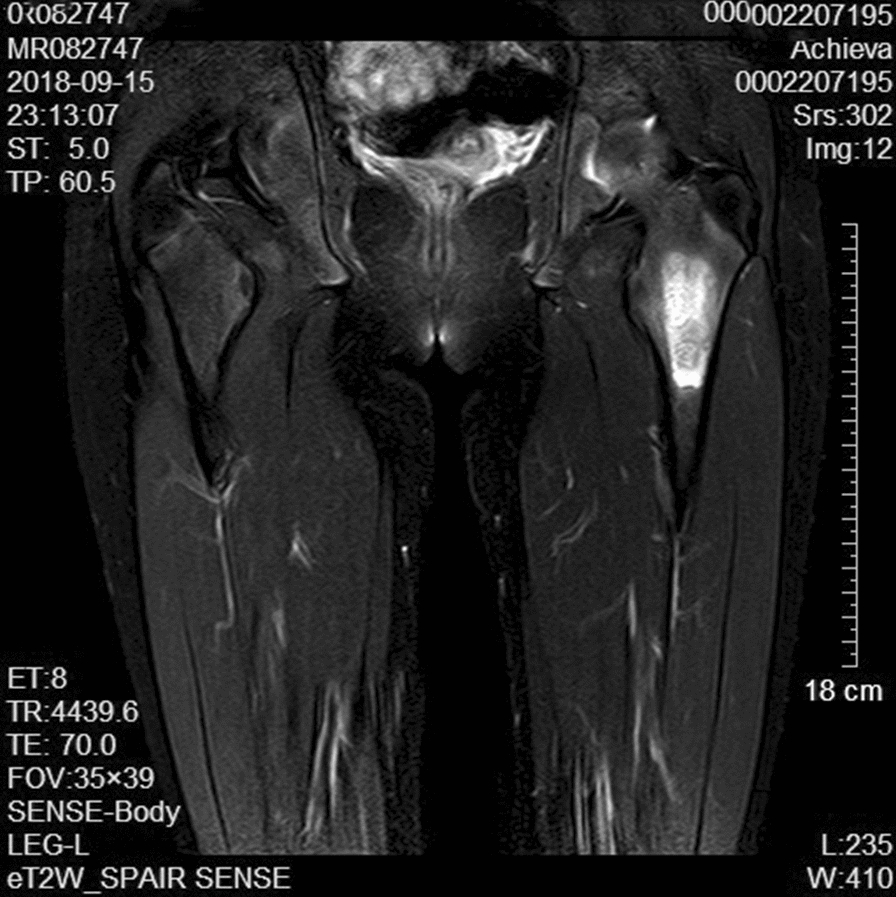
Preoperative magnetic resonance imaging (MRI) showed an oval mass that was hypointense in the T1-weighted image and hyperintense in the fat-suppressed T2-weighted image in the bone marrow cavity of the proximal femur

Physical examination at admission revealed firm, tender mild swelling of the left proximal thigh, with normal overlying skin. Adduction and flexion of the left hip were limited and elicited pain. There were no other remarkable abnormalities. Plain radiographs showed increased density of the greater trochanter of the left femur (Fig. 1). CT displayed thickened cortex in the anterior surface of the left proximal femur with specks of calcification and inhomogeneity of the adjacent bone marrow cavity (Fig. 2). Preoperative magnetic resonance imaging (MRI) showed an oval mass in the bone marrow cavity of the proximal femur, which was 5.5 × 2.1 cm in size and hypointense in the T1-weighted image and hyperintense in the fat-suppressed T2-weighted image. The intertrochanteric bone had slightly sheet-like hyperintense signal on fat suppression and a sharp protrusion was seen anterior to the lesion and its wide base was connected to the bone. The adjacent soft tissue showed a long curved shadow on T2 fat-suppressed signal. The local cortical bone was interrupted. No obvious soft tissue shadow was seen (Fig. 3). Considering that pain was the predominant symptom in the patient and the fact that osteoid osteoma is more common, especially in the proximal femur [1], based on the radiological (X-ray, CT and MRI) findings, a diagnosis of left femur tumor with a high likelihood of osteoid osteoma was made.

**Fig. 4:**
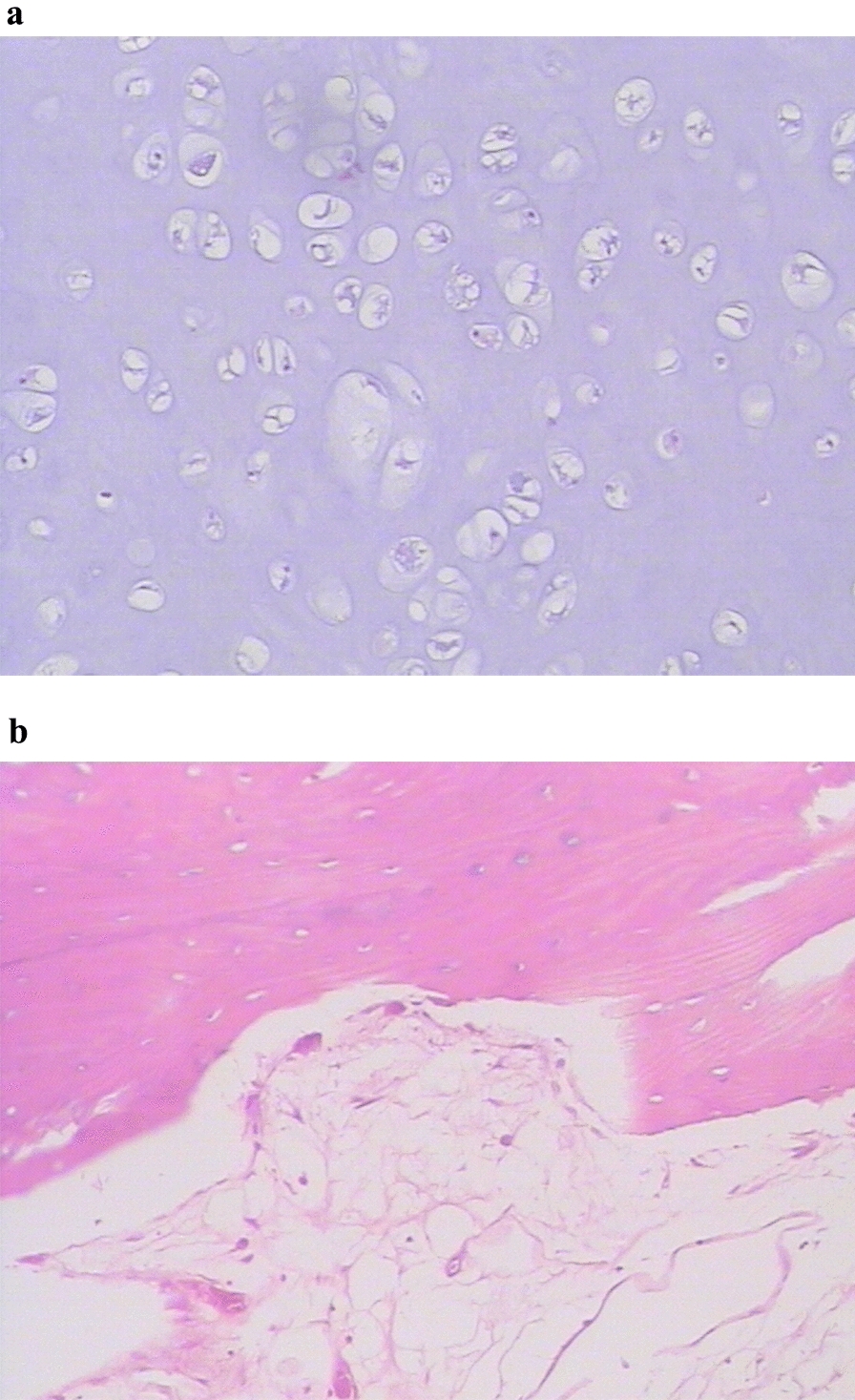
**a** A photomicrograph shows a hyaline cartilage lobule with overall homogenous, low cellularity. **b** The presence of benign-appearing chondrocytes. Chondrocytes have enlarged nuclei and irregular in shape. The adjacent cortex becomes thickened and the trabeculae are disorderly in arrangement

The patient underwent surgical exploration. Soft tissue swelling was observed adjacent to lesion in the proximal femur and massive granulation tissues were present in the medullary canal. The periosteum, and the granulation tissue and cartilage tissue were excised by curettage and pathological study revealed thickening of the periosteum and bone (Fig. 4). Iliac crest grafting in the femur lesion was performed. The surgery was uneventful and postoperative X-ray examination showed a hypodense area in the left proximal femur. A final diagnosis of enchondroma of the proximal femur was made. The patient received conventional postoperative care and was discharged from the hospital 16 days after surgery. The patient received follow-up at the hospital at 1, 3, 5 and 18 months postoperatively. At the last follow-up visit, the left hip showed good range of motion including flexion, adduction and medial and lateral rotation. The patient could ambulate without difficulty. Signs of left femoral nerve injury disappeared. Roentgenography showed irregular patchy inhomogeneous hypodense shadows with an indistinct border. No other abnormalities were seen. The imaging and pathological findings of the patient are shown in Table 1.

**Table 1:** Imaging and pathological features of the patient

	Findings
Roentgenography	Increased density of the greater trochanter of the left femur (Fig. 1)
CT	The cortex was thickened, with a size of 6.4 × 1.7 cm, in the anterior surface of the left proximal femur with specks of calcification and inhomogeneity of the adjacent bone marrow cavity. Cord-like and patchy hyperintense shadows were present. No apparent abnormality in adjacent oft tissues. The findings were highly suggestive of left femur tumor with a high likelihood of osteoid osteoma (Fig. 2)
MRI	A hypointense oval mass, 5.5 × 2.1 cm in size, was seen in the bone marrow cavity of the proximal femur in the T1-weighted image. The mass was 5.47 × 2.14 cm in size and hyperintense in the fat-suppressed T2-weighted image. The intertrochanteric bone had slightly sheet-like hyperintense signal on fat suppression and a sharp protrusion was seen anterior to the lesion and its wide base was connected to the bone. The adjacent soft tissue showed a long curved shadow on T2 fat-suppressed signal (Fig. 3)
Pathology	The medullary cavity was mainly composed of lobulated, well differentiated cartilage. The cellular density increased in the lesion, the nuclei of chondrocytes appeared large and irregular. Varying degrees of sclerotic bone reaction surrounded the lesion, and the trabeculae were in disarray with fibrous connective tissue overgrowth. Newly formed trabeculae were seen, and the soft tissues adjacent to the femur were hyperemic with focal bleeding. The vessels of small vessels became thickened and the amount of fibrous connective tissue increased with infiltration of inflammatory cells. Mucoid degeneration and muscle atrophy were present (Fig. 4)
Electromyography	Injury of the left femoral nerve
Intraoperative findings	Soft tissue swelling was observed adjacent to the lesion in the proximal femur. The periosteum became thickened and the bone in the lesion site was thickened and rough. The lesion was excised for pathological examination. Massive granulation tissues were present in the medullary canal
Follow-up	The patient received follow-up at the hospital at 1, 3, 5 and 18 months postoperatively. The left hip showed a good range of motion including flexion, adduction and medial and lateral rotation. The patient could ambulate without difficulty. Signs of left femoral nerve injury disappeared. Roentgenography showed irregular patchy inhomogeneous hypodense shadows with an indistinct border. No other abnormalities were seen

## Discussion and conclusions

Enchondromas originating in the epiphyses of the femur are very rare. In the current report, we describe a case of epiphyseal enchondroma of the proximal femur in a 15-year-old girl that was erroneously diagnosed as osteoid osteoma. Both conditions rarely occur in the epiphysis of the femur and have clinical and radiological manifestations that make diagnosis elusive. Lack of thorough understanding of the two disease entities, especially in an atypical location, indefinite radiological findings and misleading clinical and radiological features of the current case led to our erroneous diagnosis of the disease.

Osteoid osteoma is a common benign tumor among young boys and adolescents [1, 7], typically involving those aged 7–25 years, an age range which our case also falls into. Unlike metadiaphyseal enchondromas, pain is common in enchondromas originating in the epiphyses of the femur. The current patient sought medical attention because of painful leg and an osteolytic nidus was demonstrated on bone CT scan, which led us to mistakenly diagnose the disease as osteoid osteoma. We failed to consider subtle signs in the patient: pain the lower extremity differs from pain in osteoid osteoma which occurs adjacent to the lesion and does not radiate distally. The presence of injury of the left femoral nerve, not enchondroma, in the girl may be the cause of lower extremity pain while tenderness near the enchondroma may be due to osteolytic lesions in enchondroma.

The osteolytic lesions of enchondromas manifest as local bone destruction on radiographs, with a well demarcated border and specks of calcification [1]. Enchondromas may show singular or multiple niduses on bone CT scan while MRI can clearly reveal the extent of the nidus, edema and changes in adjacent soft tissue. Calcified lesions and interchondral gaps manifest as hypointense signals on T2W1. Hyperintense signals on T1W1 suggest presence of fat tissues in the tumor [1]. MRI showed an oval mass that was hypointense in the T1-weighted image and hyperintense in the fat-suppressed T2-weighted image in the bone marrow cavity of the proximal femur. Osteoid osteoma relies on the demonstration of an osteolytic nidus radiologically [1] and if there are calcification and reactive sclerosis surrounding the nidus, a characteristic sign of the owl’s eye may be present [1]. The nidus in osteoid osteoma may be very small in the early stage of the disease and may not be picked up by X-ray examination. CT scan is the best method for detecting the presence of a nidus and can also reveal the size and location of the nidus. MRI may clearly show the nidus of osteoid osteoma and adjacent edema and delineate the relation of osteoid osteoma with its adjacent anatomic structures.

Osteosarcoma, clear cell chondrosarcoma, chondroblastoma, giant cell tumor are often emphasized as differential diagnoses of lesions in the epiphysis [8] as epiphyseal enchondroma has overlapping radiologic features with clear cell chondrosarcoma, epiphyseal chondroblastoma, and epiphyseal osteosarcoma. Osteoid osteoma is often not considered in the diagnosis of epiphyseal enchondroma as both conditions rarely occur in the epiphyseal region. Our case highlights that diagnosis can become elusive when enchondromas or osteoid osteomas occur in atypical locations and present with nonspecific clinical and imaging characteristics and enchondroma should be considered in lesions of the epiphysis.

## Data Availability

Not applicable.

## Abbreviations

MRI: Magnetic resonance imaging
MRI: Magnetic resonance imaging
