# Correction to: Epiphyseal enchondroma masking as osteoid osteoma: a case report

**DOI:** 10.1186/s40001-021-00525-7

**Published:** 2021-06-13

**Authors:** Xuyang Cao, Qiang Ren, Xiangnan Li, Yiren Tian, Zhendong Wang

**Affiliations:** 1 Department of Orthopedics, General Hospital of Jizhong Energy Xingtai Mining Group, Xingtai, 054000 Hebei China; 2 Department of Orthopedics, Hebei Provincial Children’s Hospital, No. 133 Jianhua South Street, Chang’an District, Shijiazhuang, 050000 Hebei China; 3 Department of Orthopedics, Hebei Provincial People’s Hospital, Shijiazhuang, 050000 Hebei China; 4 Department of Rehabilitation, Hebei Provincial Academic of Traditional Chinese Medicine, Shijiazhuang, 050000 Hebei China

## Correction to: Eur J Med Res (2021) 26:42 https://doi.org/10.1186/s40001-021-00504-y

In the original publication of the article, the third author’s affiliation was published incorrectly. The corrected affiliation is given in this Correction article.
